# Collapse dynamics of spherical cavities in a solid under shock loading

**DOI:** 10.1038/s41598-020-64669-y

**Published:** 2020-05-21

**Authors:** E. M. Escauriza, J. P. Duarte, D. J. Chapman, M. E. Rutherford, L. Farbaniec, J. C. Jonsson, L. C. Smith, M. P. Olbinado, J. Skidmore, P. Foster, T. Ringrose, A. Rack, D. E. Eakins

**Affiliations:** 1 Department of Engineering Science, University of Oxford, Oxford, OX1 3PJ UK; 2 ESRF – The European Synchrotron, CS40220, F-38043 Grenoble, France; 3 Department of Physics, Imperial College London, London, SW7 2BZ UK; 4 First Light Fusion Ltd., Yarnton, Kidlington OX5 1QU UK

**Keywords:** Mechanical engineering, Condensed-matter physics, Applied optics, Condensed-matter physics

## Abstract

Extraordinary states of highly localised pressure and temperature can be generated upon the collapse of impulsively driven cavities. Direct observation of this phenomenon in solids has proved challenging, but recent advances in high-speed synchrotron radiography now permit the study of highly transient, subsurface events in real time. We present a study on the shock-induced collapse of spherical cavities in a solid polymethyl methacrylate medium, driven to shock states between 0.49 and 16.60 GPa. Utilising multi-MHz phase contrast radiography, extended sequences of the collapse process have been captured, revealing new details of interface motion, material failure and jet instability formation. Results reveal a rich array of collapse characteristics dominated by strength effects at low shock pressures and leading to a hydrodynamic response at the highest loading conditions.

## Introduction

The importance of the cavity collapse phenomenon was first recognised by Lord Rayleigh, among others, who in 1917 attempted to explain the progressive damage experienced by screw propellers in water. By assuming the cavity is sufficiently far from the propeller surface, Rayleigh analytically modelled a spherical void collapsing under hydrostatic pressure. The results revealed an extraordinary increase in pressure at the instant of collapse, far in excess of the strength of materials used at the time^1^. Subsequent investigations revealed that, if the collapse occurs near a solid boundary, a jet forms which impacts the solid boundary^2,3^. For both the jetting and non-jetting cases, deterioration of the propeller surface is caused by the accumulation of the actions of many individual collapsing cavities. In the modern context, cavity collapse plays an important role in several applications, including the *in-situ* destruction of kidney and gall stones through shock lithotripsy^4,5^, targeted drug delivery^6–8^, and the cleaning of surfaces exposed to ultrasonic fields in liquids^9–11^.

More recently, there has been interest in the collapse of cavities in solid media, particularly in relation to hot spot formation and ignition mechanisms in energetic materials^12–14^. Although more stable under ambient fluctuations, these cavities may be driven to collapse under intense pressure waves, such as those produced under shock loading. Depending on the magnitude of the shock relative to the strength of the solid, the dynamics of the collapse process vary significantly. For the strongest shocks, hydrodynamic collapse is expected^15^, and energy is localised during the formation of a high-speed jet and toroidal vortex that forms after jet impact^16^. Other mechanisms become dominant at lower shock pressures, when the deviatoric strength of the solid medium is significant relative to the stresses generated during the impact^15^. This also corresponds to an increase in the complexity of the collapse process, due to presence of failure and other strength-related phenomena. Simulations have been performed to model cavity collapse in a solid, in both the hydrodynamic^17,18^ and strength^19,20^ shock pressure regimes, but the precise effect that strength has on the collapse process and the mechanisms of energy dissipation is not known.

While hydrodynamic shock-induced cavity collapse has been observed experimentally in gaseous^16,21,22^ and liquid media^23–25^, direct observation in solid media has thus far proved challenging, primarily due to the opacity of most solids to visible light. Even for optically transparent solids, large refractive effects coupled with severe deformations in shock loading serve to either distort or obscure entirely details of the evolving sub-surface interfaces. A recent optical imaging study by Ma *et al*.^26^, looking at weak shock-cavity interactions in polymethyl methacrylate (PMMA), partly addressed this issue by cutting cylindrical holes from blocks of PMMA. This provided a two-dimensional (2D) cavity geometry that avoided the issues of optical refraction. Comparisons to 2D simulations gave predictions for the temperature field around the cavity during hot spot formation. Stronger shock loading, sufficient to induce collapse of a solid cavity, has yet to be observed.

For the probing of 3D cavities fully embedded in a solid, X-ray imaging (XRI) offers distinct advantages, as the higher transmission of X-rays provides direct access to the sub-surface details of the collapse process. Furthermore, compared to high-speed optical imaging, XRI benefits from reduced spatial distortion due to the negligible refraction of X-rays. Recent advancements in XRI techniques at synchrotron facilities mean it is now possible to image the sub-surface dynamics of materials at ultra-high-speed (UHS) rates of over 1 million frames per second (Mfps), with each image created from a single 100 ps X-ray pulse^27–33^. These techniques have been used to study shockwave dynamics and jet formation in shock-compressed polymer lattices by Branch *et al*.^34^, and shock-induced collapse of hollow spherical glass cavities in silicone and TNT by Armstrong *et al*.^35^. The latter study found evidence of higher temperatures in the collapsed cavity region. However, due to the sub-millimetre cavity sizes and constraints imposed by the electron bunch spacing of the synchrotron, only a single frame could be captured per collapse.

Motivated by the dearth of currently available experimental data — in both the hydrodynamic and strength regimes — this study presents direct observations of shock-cavity interactions in a solid medium. Single-stage and two-stage gas gun plate impact experiments were performed at the European Synchrotron Radiation Facility (ESRF) to generate planar shocks in PMMA targets containing spherical cavities. A bespoke UHS XRI system was employed to capture dynamic radiographs of collapsing cavities at rates exceeding 1 Mfps^32^. Fluid-dominated dynamics were studied, as well as the previously unobserved strength-dominated and transition regimes, by exploring a wide range of stress states.

## Method

### Gas gun systems

The experiments were performed at beamline ID19 of the ESRF synchrotron. A range of dynamic stress states were investigated by employing both a single-stage and two-stage gas gun, producing shock pressures from 0.49 to 16.60 GPa. Dynamic compression at beamline ID19 is a recent development, and this work presents the first use of a two-stage gas gun at the facility. As such, the experimental setup is described here in full.

**Figure 1:**
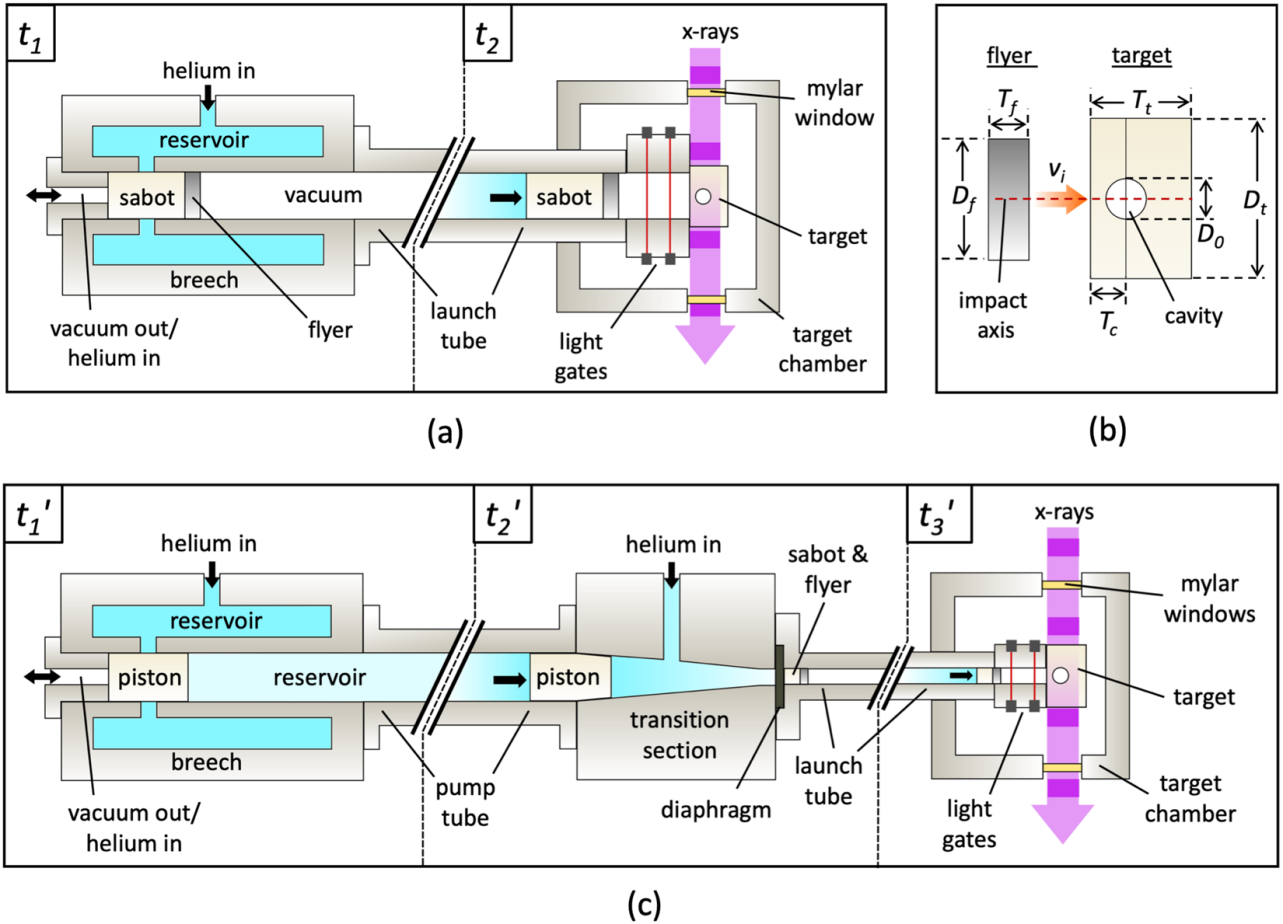
Schematic diagrams showing the setup for the experiments. In (**a**), for the single-stage experiments, the projectile is accelerated toward the target under the action of a single high-pressure reservoir. (**b**) An enlarged view of the flyer plate and target, which have cylindrical symmetry about the axis of impact. (**c**) For the two-stage experiments, high-pressure gas accelerates a piston towards a transition section, compressing and heating the helium gas ahead of it. This gas subsequently bursts a diaphragm, launching a sabot toward the target mounted at the end of the muzzle. Both gun systems are oriented normal to the X-ray beam to capture the transverse profile of the shock wave and cavity.

A schematic diagram of the single-stage gun system can be seen in Fig. 1(a) (based on the system used in earlier experiments by Eakins and Chapman^29^). The apparatus consists of a breech and target chamber, connected by a 25 mm bore barrel. A projectile (consisting of a sabot fronted by a flyer plate) is accelerated down the barrel with high pressure helium gas initially contained within the breech (*t*_1_). After the projectile is launched from the breech (*t*_2_), a pair of light gates records its velocity before it reaches the target, which is mounted within the evacuated target chamber. These light gates also serve to trigger the imaging system. The entire gun apparatus is oriented to place the impact axis perpendicular to the propagation direction of the synchrotron X-rays, giving a side-on radiographic projection of the event. Low-Z Mylar windows in the target chamber maintain the vacuum whilst minimising X-ray attenuation.

Figure 1(c) shows the two-stage gas gun setup, which has an additional transition section, connecting a 50 mm bore pump tube to the second ‘stage’ of the gun, a 10 mm bore launch tube. Prior to firing a piston is loaded into the breech and a projectile (sabot and flyer plate) is loaded into the launch tube. The breech is charged with helium and the pump tube evacuated then filled with helium at near atmospheric pressure (${t}_{1}{\rm{{\prime} }}$). Upon firing, the piston is accelerated from the breech, rapidly compressing the helium in the pump tube into the transition section (${t}_{2}{\rm{{\prime} }}$). The transition section is sealed with a diaphragm, which ruptures at a desired pressure, allowing helium (at a pressure of multiple kbar) to flow into the 10 mm bore launch tube. This accelerates the projectile toward the target, again held under vacuum within the target chamber (${t}_{3}{\rm{{\prime} }}$).

### Flyer and target design

A diagram of the flyer impacting the target is shown in Fig. 1(b). The targets were constructed from two PMMA cylinders glued together, each with a centred hemisphere cutout, forming a single cylinder containing a spherical cavity. For the single-stage gas gun experiments, the flyer plates were 25 mm diameter aluminium 2024, and were launched at velocities ranging from 0.17 to 0.80 km/s, generating shock pressures ranging from 0.49 to 2.44 GPa. Polycarbonate flyer plates 9 mm in diameter were used during the two-stage experiments, fired at impact velocities ranging from 1.70 to 4.70 km/s, producing shock pressures ranging from 4.80 to 16.60 GPa. The design of the flyers and targets ensured cylindrical symmetry upon impact (assuming negligible tilt between the flyer and target). Cavity diameters of 3, 4 and 6 mm were used for the experiments. Two of the single-stage impacts were performed in reverse, by mounting the cavity target onto a sabot and impacting it against a stainless steel anvil. In doing so, intermediate shock pressures of 2.72 GPa and 3.08 GPa were achieved that were not possible with the standard single- and two-stage impact configurations.

### Imaging systems

**Figure 2:**
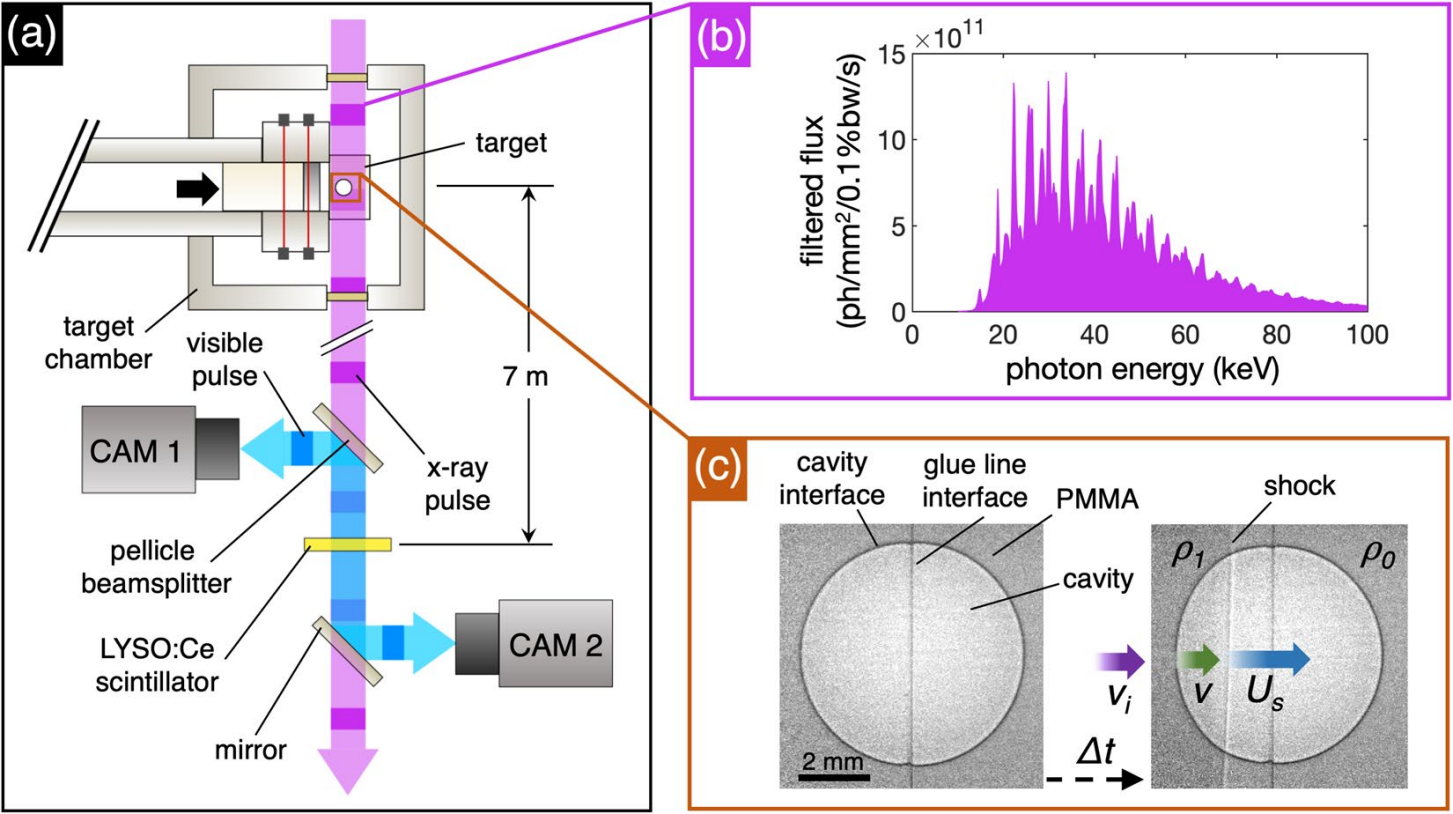
Illustration showing key features of the experiment. (**a**) Schematic diagram showing the imaging system, which is placed 7 m away from the target chamber. The X-ray pulses are absorbed by a LYSO:Ce scintillator after passing through the experiment, which re-emits visible photons. The front- and rear-surface scintillator emissions are folded away from the X-ray beam path by a mirror and pellicle beamsplitter, respectively, and are captured by two UHS cameras. (**b**) Model of the photon flux (ph/mm^2^/0.1%bw/s) with respect to photon energy (keV) of ESRF beamline ID19, calculated in XOP^66^. (**c**) Radiographs of a PMMA target containing a spherical cavity. The left image shows the stationary cavity before impact. The right image shows the target after impact, with the velocities and densities given.

Real-time UHS XRI was performed using the ESRF’s 16-bunch filling mode, which provides a continuous train of ~100 ps X-ray pulses separated by 0.176 μs. The maximum frame-rate of a radiograph sequence is thus limited by this inter-pulse time. A plot of the undulator X-ray source spectrum can be seen in Fig. 2(b), showing the X-ray spectral flux at the sample in photons/mm^2^/0.1%bw/s as a function of the photon energy in keV. The softer X-rays below 10 keV were absorbed by diamond and aluminium filters, reducing the heat load on the scintillator and optics. The result is a mean absorbed X-ray energy of ~30 keV. Based on the camera resolution and the size of the features of interest, the imaging system was placed 7 m downstream from the experiment such that the radiographs were recorded in the near-field Fresnel diffraction regime. Such a standoff was sufficient to introduce ‘phase contrast’ interference fringes in the radiographs, which are pronounced at positions where there is a sudden change in the refractive index of the material. The result is enhanced contrast of material edges^36,37^, which allows for the *in-situ* tracking of cavity collapse features, such as the cavity interface position, shock position and new interfaces (cracks).

A schematic diagram of the imaging system can be seen in Fig. 2(a). X-rays transmitted through the sample are indirectly imaged using a 250 μm thick LYSO:Ce scintillator (Ce-doped Lu_1.8_Y_0.2_SiO_5_, Hilger Crystals, UK), which absorbs X-ray photons and re-emits visible photons at a peak wavelength of 420 nm^38^. The visible light emitted both down- and up-range of the scintillator is folded away from the X-ray beam path by a pellicle beam splitter and mirror, prior to being captured by two Shimadzu Hyper Vision HPV-X2 cameras. The HPV-X2 has a CMOS burst image sensor in a (250 × 400) pixel array format with 32 × 32 μm pixel size. The lens system provided a 1:1 magnification, producing a 8 × 12 mm field of view. For the single stage experiments, one HPVX-2 camera was used to record every third X-ray bunch, giving an inter-frame time of 0.528 μs and frame-rate of 1.89 Mfps. This three-bunch minimum frame separation time was imposed by maintaining synchronisation of the camera exposure and X-ray bunch structure. Two cameras were used for the two-stage experiments, with the images interleaved to produce a single image sequence with an effective frame rate of 3.79 Mfps for images separated by 0.176 and 0.352 μs. A more detailed explanation of the imaging system, including further modifications to include a third camera and second in-line scintillator, is given by Escauriza *et al*.^32^.

Optical imaging of the experiments was also performed to reveal the stages of tori formation during cavity collapse in the high-pressure regime. An Invisible Vision UHSi-24 framing camera was employed for this purpose, imaging the rear of the target (opposite the impact) using a mirror. The rear surface of the PMMA target was polished to allow capture of the toroidal plasma emission arising from rapid compression of trapped gas inside the collapsing cavity.

### Hydrodynamic simulations

Two-dimensional axisymmetric simulations of the experiments were performed in Hytrac, a multi-material front-tracking Eulerian hydrocode which has been used in earlier studies to model dynamic flow instabilities in PMMA under shock compression^39^. As the code does not include material strength, these simulations provided a baseline understanding of the collapse within a fluid medium, allowing the effects of strength to be more clearly contrasted to fully hydrodynamic behaviour. Hytrac is based on an earlier front-tracking code *FronTier*^40^, which has been successfully applied to shock-cavity interactions in a liquid, in both 2D and 3D^41,42^. In the present work, a cell-based adaptive mesh refinement (AMR) scheme was used to balance computational resources with resolution requirements.

For the PMMA targets and flyer materials a Frankfurt equation of state package was used to relate the pressure and specific internal energy to the density and temperature, producing a tabulated equation of state^43^. Two sets of simulations were performed, for the single-stage and two-stage experiments, with initial cell sizes of 100 μm and 137.5 μm, respectively. For both sets, 4 levels of AMR were used, giving minimum cell sizes one eighth of the initial value. An axisymmetric half-domain was modelled with a central reflective boundary and transmissive boundaries along the other three edges. For the simulations of the two-stage experiments the whole flyer and target geometries were simulated because of the small size of the target and flyer. This ensured that effects from rarefaction waves at the edge of the projectile were accounted for. Due to the larger target and flyer sizes used in the single-stage experiments, release effects were not a concern. This allowed the simulation setup to simply be a uniform domain of PMMA with a central cavity located a fixed distance from the loading surface. To replicate the impact event, the loading surface was assigned the particle velocity corresponding to the shock state of interest.

## Results

### Shock state calculations

The pressure *p* generated upon impact is calculated from the Rankine-Hugoniot jump conditions, where the shock pressure is given by the product of the shock impedance, $Z={\rho }_{0}{U}_{s}$, and the particle velocity behind the shock, *u*_*p*_, such that,

1 $$p={\rho }_{0}{U}_{s}{u}_{p},$$

where *ρ*_0_ is the initial density of the material and *U*_*s*_ is the shock velocity^15^. From the conservation of momentum jump condition, *p* and *u*_*p*_ must be continuous at the interface between the flyer and target at the instant after impact. This allows respective values to be calculated at the intercept between the *p-u*_*p*_ Hugoniot projections for the target and flyer, with the initial particle velocity of the flyer given by the impact velocity *v*_*i*_. The values of *U*_*s*_ in Eq. 1 for each material are calculated with the empirical *U*_*s*_-*u*_*p*_ relationships found in the literature (Table 1). As the impact pressure is increased in PMMA there is a transition from a cubic to linear relationship at ${u}_{p}=0.5\,{\rm{km}}/s$^44,45^. This transition has been attributed to a change in the mechanical response of polymers under shock compression, due to a large difference between forces along the polymer chain and forces between neighbouring chains^46^.

**Table 1:** The *U*_*s*_-*u*_*p*_ Hugoniot for each material, used to calculate the shock pressure.

Material	*ρ*_0_(g/cm^3^)	Hugoniot Relation	Ref.
PMMA (*u*_*p*_ < 0.5 km/s)	1.187	${U}_{s}=9.17{{u}_{p}}^{3}-9.47{{u}_{p}}^{2}+3.64{u}_{p}+2.74$	^44^
PMMA (*u*_*p*_ > 0.5 km/s)	1.187	*U*_*s*_ = 1.54*u*_*p*_ + 2.55	^44^
Aluminium 2024	2.784	*U*_*s*_ = 1.29*u*_*p*_ + 5.37	^45^
Polycarbonate	1.193	*U*_*s*_ = 1.20*u*_*p*_ + 2.92	^45^
Stainless steel	7.910	*U*_*s*_ = 1.49*u*_*p*_ + 4.58	^45^

It is also possible to measure *U*_*s*_ and *u*_*p*_ from the shock front and flyer positions in the radiographs. However, because of the small distance between the impact surface and cavity interface and the large shock velocity relative to the inter-frame time, it is not always possible to measure the shock front displacement before it has interacted with the cavity. Furthermore, the flyer interface during the early stages of compression could not be accurately identified due to the poorer signal to noise ratio near the periphery of the radiographs. By contrast, the impact velocity *v*_*i*_ was determined to within 1% uncertainty and the equations of state given in Table 1 are well established. For these reasons, the pressures measured from the radiographs were used only as a check against the pressures calculated using *v*_*i*_ and the impedance matching method. A summary of the key experimental details, and the results of the impedance matching calculations, can be seen in Table 2. As labelled in the diagram of Fig. 1(b), *D*_0_ is the cavity diameter, *D*_*t*_ is the target diameter, *T*_*t*_ is the target thickness and *T*_*c*_ is the distance between the target impact face and the cavity centre. The uncertainties in *u*_*p*_, *U*_*s*_ and *p* were calculated from the uncertainties associated with *v*_*i*_ and the ~3% uncertainty associated with the *U*_*s*_-*u*_*p*_ equations of state for PMMA given in Table 1 ^44^.

**Table 2:** Details of the results and the three setups used for the experiments.

*D*_0_ (mm)	*D*_*t*_ (mm)	*T*_*t*_ (mm)	*T*_*c*_ (mm)	*v*_*i*_ (km/s)	*u*_*p*_ (km/s)	*U*_*s*_ (km/s)	*p* (GPa)
***Single-stage gas gun, single-camera, 25 mm flyer diameter, 5 mm flyer thickness***							
4	20	12	5	0.200 ± 0.001	0.16 ± 0.01	3.12 ± 0.10	0.60 ± 0.02
4	20	12	5	0.396 ± 0.002	0.32 ± 0.01	3.23 ± 0.10	1.21 ± 0.04
4	20	12	5	0.595 ± 0.003	0.47 ± 0.02	3.28 ± 0.10	1.86 ± 0.06
4	20	12	5	0.750 ± 0.004	0.59 ± 0.02	3.46 ± 0.11	2.44 ± 0.08
6	20	12	6	0.165 ± 0.001	0.13 ± 0.01	3.08 ± 0.10	0.49 ± 0.02
6	20	12	6	0.406 ± 0.002	0.32 ± 0.01	3.24 ± 0.10	1.25 ± 0.04
6	20	12	6	0.589 ± 0.003	0.47 ± 0.02	3.31 ± 0.10	1.84 ± 0.06
***Single-stage gas gun, single camera, reverse impact against stainless steel anvil***							
4	25	13	6	0.719 ± 0.004	0.65 ± 0.02	3.54 ± 0.12	2.72 ± 0.09
4	25	13	6	0.795 ± 0.004	0.71 ± 0.03	3.65 ± 0.11	3.08 ± 0.10
***Two-stage gas gun, two cameras, 9 mm flyer diameter, 2 mm flyer thickness***							
3	11	11	2.5	1.973 ± 0.010	0.99 ± 0.03	4.08 ± 0.13	4.80 ± 0.15
3	11	11	2.5	2.985 ± 0.015	1.48 ± 0.05	4.83 ± 0.15	8.49 ± 0.26
4	11	11	3	3.020 ± 0.015	1.50 ± 0.05	4.85 ± 0.15	8.63 ± 0.26
4	11	11	3	3.937 ± 0.020	1.94 ± 0.06	5.53 ± 0.17	12.71 ± 0.39
6	11	11	4	3.868 ± 0.019	1.90 ± 0.06	5.48 ± 0.17	12.38 ± 0.38
6	11	11	4	3.957 ± 0.020	1.95 ± 0.06	5.55 ± 0.17	12.80 ± 0.39
6	11	11	4	4.697 ± 0.023	2.30 ± 0.07	6.09 ± 0.19	16.60 ± 0.49

The values for *u*_*p*_, *U*_*s*_ and *p* were calculated using Eq. 1 and the impedance matching method.

### Experimental data

**Figure 3:**
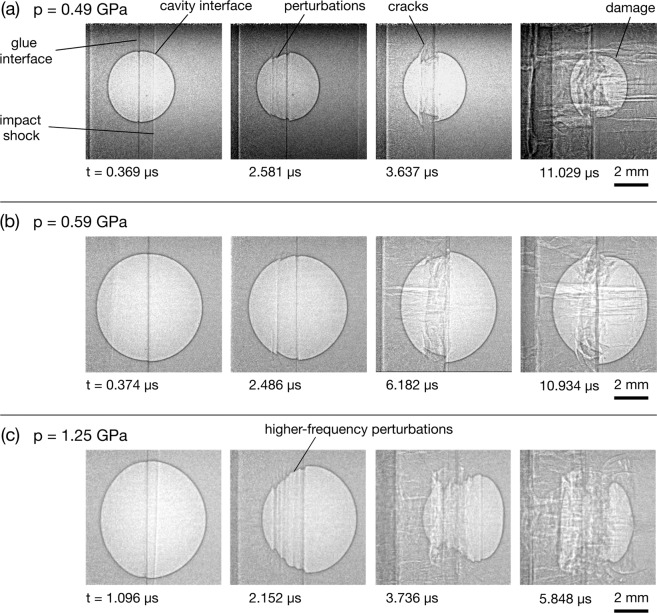
Radiograph sequences showing shock-cavity interactions in the strength-dominated regime. (**a**) A 4 mm cavity and 0.49 GPa shock. (**b**) A 6 mm cavity and 0.59 GPa shock. (**c**) A 6 mm cavity and 1.25 GPa shock.

**Figure 4:**
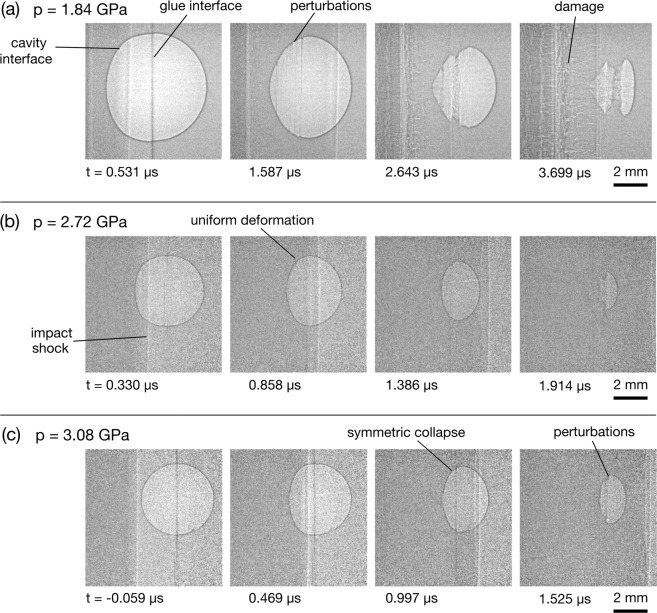
Radiograph sequences showing shock-cavity interactions in the transition regime. (**a**) A 6 mm cavity and 1.84 GPa shock. (**b**) A 4 mm cavity and 2.72 GPa shock. (**c**) A 4 mm cavity and 3.08 GPa shock.

**Figure 5:**
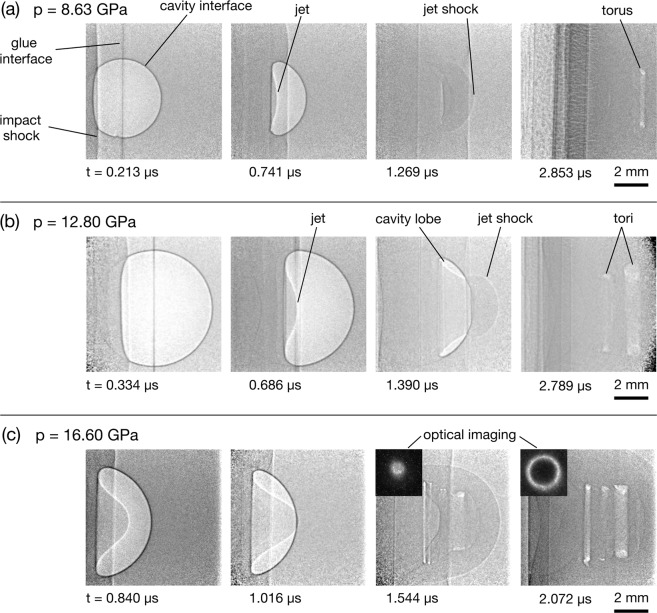
Radiograph sequences showing shock-cavity interactions in the hydrodynamic regime. (**a**) A 4 mm cavity and 8.63 GPa shock. (**b**) A 6 mm cavity and 12.80 GPa shock. (**c**) A 6 mm cavity and 16.60 GPa shock, with insets showing the rear-surface optical images of the toroidal plasma emission.

Example radiographs showing a 6 mm cavity experiment can be seen in Fig. 2(c). The left-hand image shows the cavity stationary before impact, and the right-hand image shows the early interaction between the shock and the cavity after impact. The planar shock front, which propagates at velocity *U*_*s*_, is visible as a phase contrast fringe separating the lighter uncompressed region and the darker compressed region of the target, which have densities *ρ*_0_ and *ρ*_1_, respectively. The shock releases at the free surface of the cavity interface and propagates in the direction of the shock with velocity *v* at time *t* after release. The cavity interface and glue line are also clearly visible because of the prominent dark phase contrast fringe. The projected area of the cavity is measured as the number of pixels enclosed by the cavity interface. The proportional change in area can thus be calculated as the ratio of the area in the images of the deformed cavity to the area before impact, providing a normalised and dimensionless measurement. The results of these calculations are presented in Section 3.5. Radiograph sequences from the experiments can be seen Figs. 3–5, which show shock-cavity interactions in different pressure regimes, from weak to strong shocks.

### The strength regime

Radiographs from a single-stage gas gun experiment can be seen in Fig. 3, which show the features of strength-dominated collapse dynamics. Figure 3(a) shows a 6 mm diameter cavity impacted from the left with an aluminium flyer at 0.165 km/s, producing a shock pressure of 0.49 GPa. The times given are relative to contact between the shock and the upstream cavity surface. At 0.369 μs there is visible deformation of the upstream cavity interface, with the shock front and glue line interface also visible. Perturbations and sites of crack initiation can be seen at 2.581 μs. At 11.029 μs a large amount of damage is visible, and there are axially aligned cracks. These features are qualitatively similar to the shear banding formed in the simulations of weakly shocked cavity collapse in HMX^19,20^.

The radiographs in Fig. 3(b,c) show cavities subjected to shock pressures of 0.59 GPa and 1.25 GPa, respectively. At these increased pressures the collapse behaviour is dominated by uniform deformation until later times, and the spacing of the surface perturbations that form is smaller. As can be seen in Fig. 4, this trend continues as the shock pressure is increased and the dynamics transition from a failure mode dominated by brittle fracture to one dominated by material flow. The images, which show experiments between 1.84 and 3.08 GPa, show uniform deformation dominating for a larger proportion of the collapse, with the shape similar to that of a bubble collapsing under hydrostatic compression. Note that the experiments shown in Fig. 4(b,c) are reverse impact, in which the cavity targets were impacted against a steel anvil.

### The hydrodynamic regime

The radiograph sequences in Fig. 5 show fluid-dominated collapse dynamics. In all three sequences the cavity was impacted with a polycarbonate flyer using a 2-stage gas gun. In this regime the formation of a pronounced jet can be seen, typical of strong shock-cavity interactions in gaseous and liquid media. This feature was predicted in the simulations of shock-cavity interactions in a gaseous medium^47^. Experimentally it has been observed in spherical cavities in gas^21,22^ and cylindrical cavities in hydrogel^23,24^. Pronounced jet formation was also predicted in simulations of strong shock-cavity interactions in HMX, for both cylindrical^20,48^ and spherical^18^ cavity geometries.

For all three radiograph sequences in Fig. 5, after the cavity has completely collapsed the remaining hot gas and vapourised PMMA forms one or more visible tori, which propagate downstream. The tori are formed by the deposition of vorticity along the cavity surface during the jet impact^16^. The formation of tori was observed in 3D simulations of shocks interacting with micrometre-sized spherical cavities in HMX^17,18^ and spherical helium cavities in air^49^. While experimental observation of tori formation has thus far been restricted to gaseous^21,22^ and liquid^24^ media, this work has confirmed the phenomena extends to solids subjected to strong shock compression. Through simultaneous optical imaging, the toroidal plasma emission was captured through the transparent rear surface of the target. The early and late stages of the toroid formation can be seen in the optical imaging insets in Fig. 5(c), which have been matched to the approximate time in the radiographs.

### Cavity collapse time evolution

**Figure 6:**
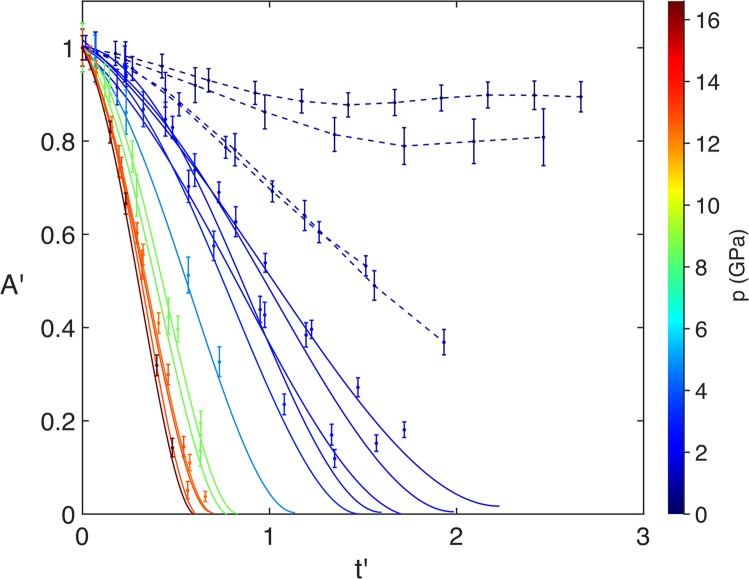
Plots of *A*′ vs. *t*′. The colour bar shows the shock pressure *p*. In the low-pressure region the dashed lines show the trajectory of the projected area, with a piecewise linear interpolation between each point. In the higher-pressure region the solid lines show the fitting of each curve with Eq. 4.

The normalised projected cavity area *A*′ = *A/A*_0_, where *A* is the area at time *t* and *A*_0_ is initial area, is a quantifiable parameter of cavity collapse. This metric has been used in both experimental^24,25^ and computational studies^50–52^, which were performed with hydrogel and water as the surrounding medium, respectively. These earlier experiments considered cylindrical cavities, in which the cross section remains constant throughout the cavity, and the volume scales with normalised cavity area. In the present study the use of 3D spherical cavities means only the maximum projected area of the cavity can be directly measured from the radiographs, as the cross-sectional area varies with depth. The measured data can be seen in Fig. 6, which shows the time evolution of *A*′, with each line representing a single experiment. The horizontal axis is presented in dimensionless time $t{\rm{{\prime} }}=t/({D}_{0}/{C}_{L})$, where *t* is in microseconds, *D*_0_ is the initial cavity diameter in millimetres and ${C}_{L}\,\mathrm{=\; 2.75}\,{\rm{mm}}/{\rm{\mu }}{\rm{s}}$ is the longitudinal sound speed in PMMA^44^. A value of *t*′ = 1 thus corresponds to the time taken for a longitudinal sound wave in PMMA to travel a distance of one cavity diameter. The time *t*′ = 0 is defined as the time at which the shock front first reaches the cavity interface. The colours in the plots represent a linear change in the initial shock pressure, from 0 to 16.6 GPa. As expected, there is a steepening of the gradient as the shock pressure increases due to the increased rate of collapse.

For cavities that underwent complete collapse (defined here as cases in which the cavity area is reduced to less than 5% of its initial value), we can define the collapse time ${t{\rm{{\prime} }}}_{col}$ as the time at which *A*′ reaches its minimum value. It was not possible to obtain the precise collapse time from the area trajectories, as the exact instant of minimum area was not captured. For the data from the two-stage experiments, this was due to the short duration of the collapse process combined with the limited frame-separation times of the image sequences from the two-camera imaging system, which are 0.176 μs and 0.352 μs. For example, the 6 mm cavity in Fig. 5(b), which was impacted with a 12.80 GPa shock, collapsed in approximately 1.5 μs. This means a maximum of five frames could be recorded during the collapse, which didn’t guarantee capturing the final stage of the collapse. Furthermore, for the single-stage experiments, the increased level of fragmentation in the PMMA medium over time meant that after a certain point it is no longer possible to quantitatively resolve the cavity interface. Therefore, for the cavities that did collapse, ${t{\rm{{\prime} }}}_{col}$ was found either by fitting the collapse curves or by estimating the collapse time from the radiographs (when fitting was not appropriate). These two processes are described later. At the two lowest shock pressures studied (0.49 GPa and 0.60 GPa) the cavities did not undergo complete collapse. Rather, failure along the cavity interface breaks up the cavity before total collapse can occur.

Complete collapse was observed at shock pressures of 1.25 GPa and above. Jet formation, jet impact and tori formation were observed at shock pressures greater than 8.49 GPa (Fig. 6). Between 1.84 GPa and 4.80 GPa, rather than forming a jet, the shape of the interface during collapse was more similar to the symmetrical shape produced by hydrostatic Rayleigh collapse. At these lower shock pressures, the pressure field forms around the cavity after the shock passes, akin to the hydrostatic pressure field in an extended fluid region. A similar result was observed in the simulations of Austin *et al*.^19^, which included the interaction between a 9.4 GPa shock and 1 μm cavity in HMX. They also noted high levels of deviatoric stresses around the cavity, which cause highly localised temperature regions in the form of shear bands. The surface perturbations and crack formation visible in Fig. 3 also suggest the presence of this type of shear localisation.

For the experiments in which total collapse was observed, a non-linear relationship between the dimensionless collapse time ${t{\rm{{\prime} }}}_{col}$ and the shock pressure *p* was observed. Such non-linearity was predicted in the analysis of Rayleigh collapse, which showed the dimensionless collapse time scales inversely with the square root of the pressure difference between the fluid pressure in the liquid medium far from the cavity and the vapour pressure of the gas inside the cavity^1,53^. A similar scaling is found for the hydrostatic collapse of a cavity near a solid boundary, with an additional prolongation factor to account for the non-spherical collapse^54^. An inverse square root dependency has also been found for numerical simulations of shock-induced collapse, showing remarkable similarities between Rayleigh collapse and shock-induced collapse, despite their notable differences^51,55^. However, a deviation from this exponent was found by Swantek & Austin, who looked at the collapse times of shock and stress wave induced hydrodynamic collapse of cylindrical cavities in agarose-GGB gel^25^. As in the present study, millimetre-sized cavities were used. Including data from a similar Dear & Field study^56^, they found that the dimensionless minimum volume time against the pressure ratio *p*/*p*_0_, where *p*_0_ is the initial pressure of the surrounding medium, follows the power-law relationship,

2 $${t{\rm{{\prime} }}}_{col,SA}=305{\left(\frac{p}{{p}_{0}}\right)}^{-0.55},$$

where ${t{\rm{{\prime} }}}_{col,SA}$ is the time to minimum volume found by Swantek & Austin^25^.

In the present work, the dimensionless collapse time ${t{\rm{{\prime} }}}_{col}$ is determined from the plots in Fig. 6. As the exact collapse time for each cavity was not known, it was necessary to fit a function to each collapse curve to find the time of minimum area ${t{\rm{{\prime} }}}_{col}$. A functional form was chosen with the boundary conditions that the area is initialised at a normalised value of 1, and decreases according to a square dependence on an effective radius, r*,

3 $$A{\rm{{\prime} }}={r}^{\ast 2}+c.$$

The constant *c* allows for cases when the area does not completely fall to zero. After comparing functional forms to hydrocode simulations run in Hytrac, the effective radius that most closely matched the simulation data was ${r}^{\ast 2}=(1-at{\rm{{\prime} }}-b{t{\rm{{\prime} }}}^{2})$, where *a*, *b* and *c* were fitting parameters, yielding the following expression for normalised projected area,

4 $$A{\rm{{\prime} }}=(1-at{\rm{{\prime} }}-b{t{\rm{{\prime} }}}^{2})+c.$$

**Figure 7:**
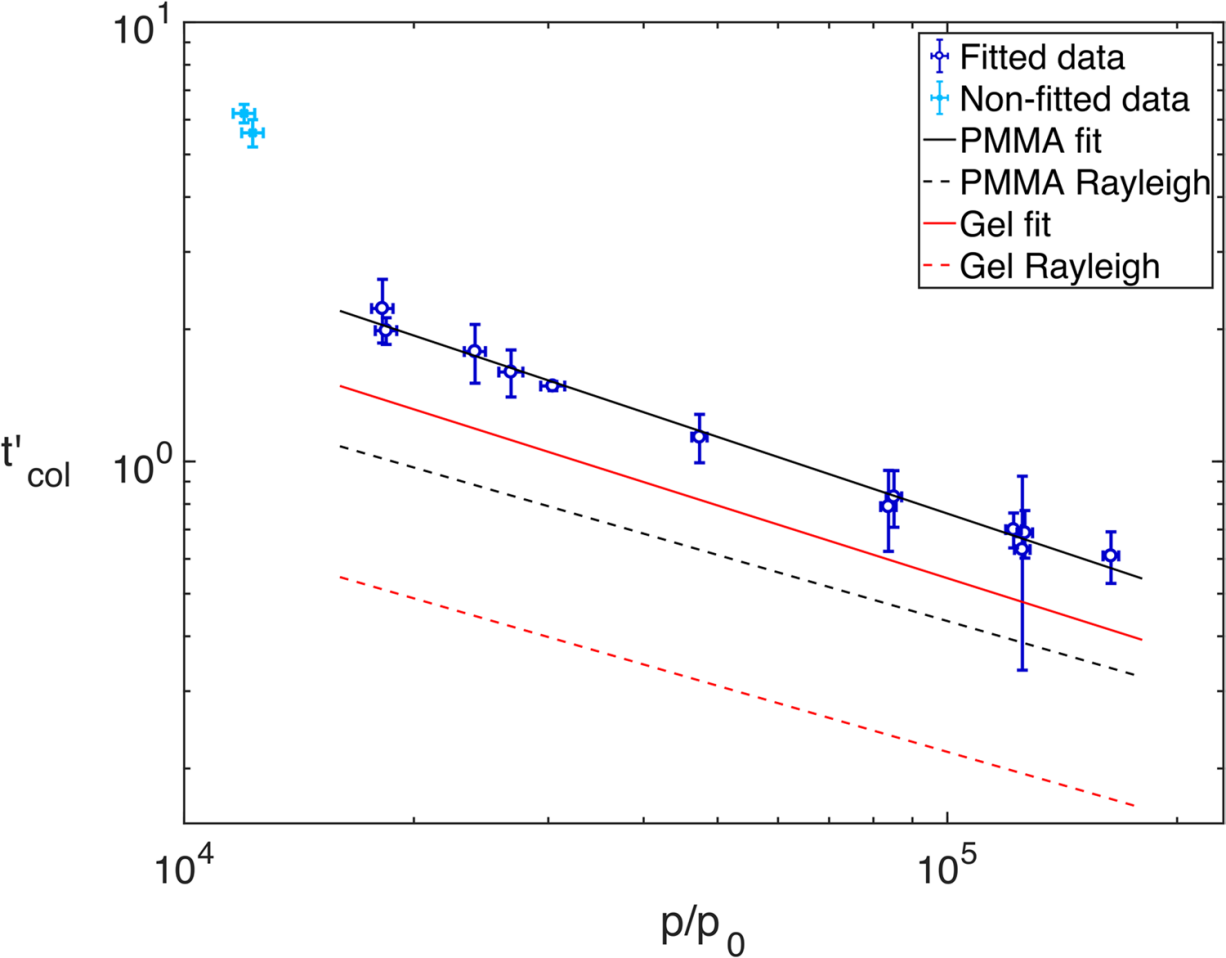
Logarithmic plot of ${t{\rm{{\prime} }}}_{col}$ vs. *p*/*p*_0_ for shock-induced and Rayleigh collapse. The open circles show the collapse times obtained from curve fitting and the closed symbols show the collapse times estimated from the radiographs. The solid black line shows the fit for the data, with *p* > 1.25 GPa, ${t{\rm{{\prime} }}}_{col}=617{(p/{p}_{0})}^{-0.58}$, and the solid red line shows the fit from the Swantek & Austin study, ${t{\rm{{\prime} }}}_{col,SA}=305{(p/{p}_{0})}^{-0.55}$^25^. The dashed black and red lines show the respective theoretical Rayleigh curves for PMMA, ${t{\rm{{\prime} }}}_{col,PMMA}^{R}=137{(p/{p}_{0})}^{-0.5}$, and water, ${t{\rm{{\prime} }}}_{col,gel}^{R}=69{(p/{p}_{0})}^{-0.5}$.

For two of the experiments the cavities did not collapse. These can be seen as the two dashed lines in the top of the figure that increase in area after reaching a minimum. For the next two highest pressures, although the cavities did collapse, the functional form did not agree with the collapse times observed in the radiographs. These curves, which show the 1.21 GPa and 1.25 GPa experiments, are shown as the two blue dashed lines in the middle of Fig. 6. The collapse times were instead estimated from the radiographs. For all the fitted curves, shown as solid lines in the figure, the collapse times agreed well with the apparent collapse times observed from the radiographs. Using a *p*_0_ value of $1\,{\rm{atm}}=1.01\times {10}^{5}\,{\rm{Pa}}$ to determine the pressure ratio, the power-law relationship is determined from a linear fit of the *p*/*p*_0_ vs. ${t{\rm{{\prime} }}}_{col}$ logarithmic plot, which is shown in Fig. 7. Rearranging the logarithmic form gives,

5 $${t{\rm{{\prime} }}}_{col}=617{\left(\frac{p}{{p}_{0}}\right)}^{-0.58},$$

with an *R*^2^ value of 0.99 determined by the fitting. The two closed-symbol points in the top left of the figure show the collapse times estimated from the radiographs, for the 1.21 GPa and 1.25 GPa experiments, which were not used in the power-law fitting. This behaviour away from the power law is possibly explained either by a change in failure mode from cracking to flow, or because of release effects at later times. In terms of the power relationship, this is in strong agreement with the Swantek & Austin finding in Eq. 2 ^25^. This is perhaps unexpected given the differences between the experimental arrangements: in the present study spherical cavities were used, as opposed to cylindrical; the pressure ratios explored are an order of magnitude greater; and the initial state of the cavity medium is a solid, rather than a liquid. The closely matched exponents thus suggest a universal mechanism underlying the collapse of cylindrical and spherical cavities, in both liquid and solid media.

Also shown in Fig. 7 are dashed black and red lines, showing the collapse time vs. pressure ratio for Rayleigh collapse in PMMA and water respectively. The Rayleigh model^1,53^, which applies to the hydrostatic compression of a bubble in an infinite fluid, predicts a collapse time based on the density *ρ* and pressure *p* of the fluid, given by,

6 $${t}_{col}^{R}=0.4573{D}_{0}\sqrt{\rho /p}.$$

To plot the dimensionless Rayleigh collapse time, the same scaling was used as for the data, such that,

7 $${t{\rm{{\prime} }}}_{col}^{R}={t}_{col}^{R}/({D}_{0}/{C}_{L})=0.4573{C}_{L}\sqrt{\rho /p}.$$

This gives respective expressions for PMMA and agarose-GG gel of,

8 $${t{\rm{{\prime} }}}_{col,PMMA}^{R}=137{(p/{p}_{0})}^{-0.5}$$

and

9 $${t{\rm{{\prime} }}}_{col,gel}^{R}=69{(p/{p}_{0})}^{-0.5}.$$

Despite the strong agreement in the exponents of experimental scaling laws for the present work and the Swantek & Austin study, there is a significant offset between the two lines, caused by a large difference in their multiplicative factors. Interestingly, for both shock-induced collapse (solid lines) and Rayleigh collapse (dashed lines), the same ratio is observed between the factors for PMMA and the gel: 617/305 = 137/69 = 2.0. This ratio can be explained by the difference in sound speed and density of the two materials, which are used to calculate the scaled collapse time for the Rayleigh expressions: ${C}_{{L}_{PMMA}}\sqrt{{\rho }_{PMMA}}/{C}_{{L}_{gel}}\sqrt{{\rho }_{gel}}=2.0$, where the gel sound speed has been taken from Swantek & Austin and the gel density is assumed to be that of water.

### Cavity interface dynamics

**Figure 8:**
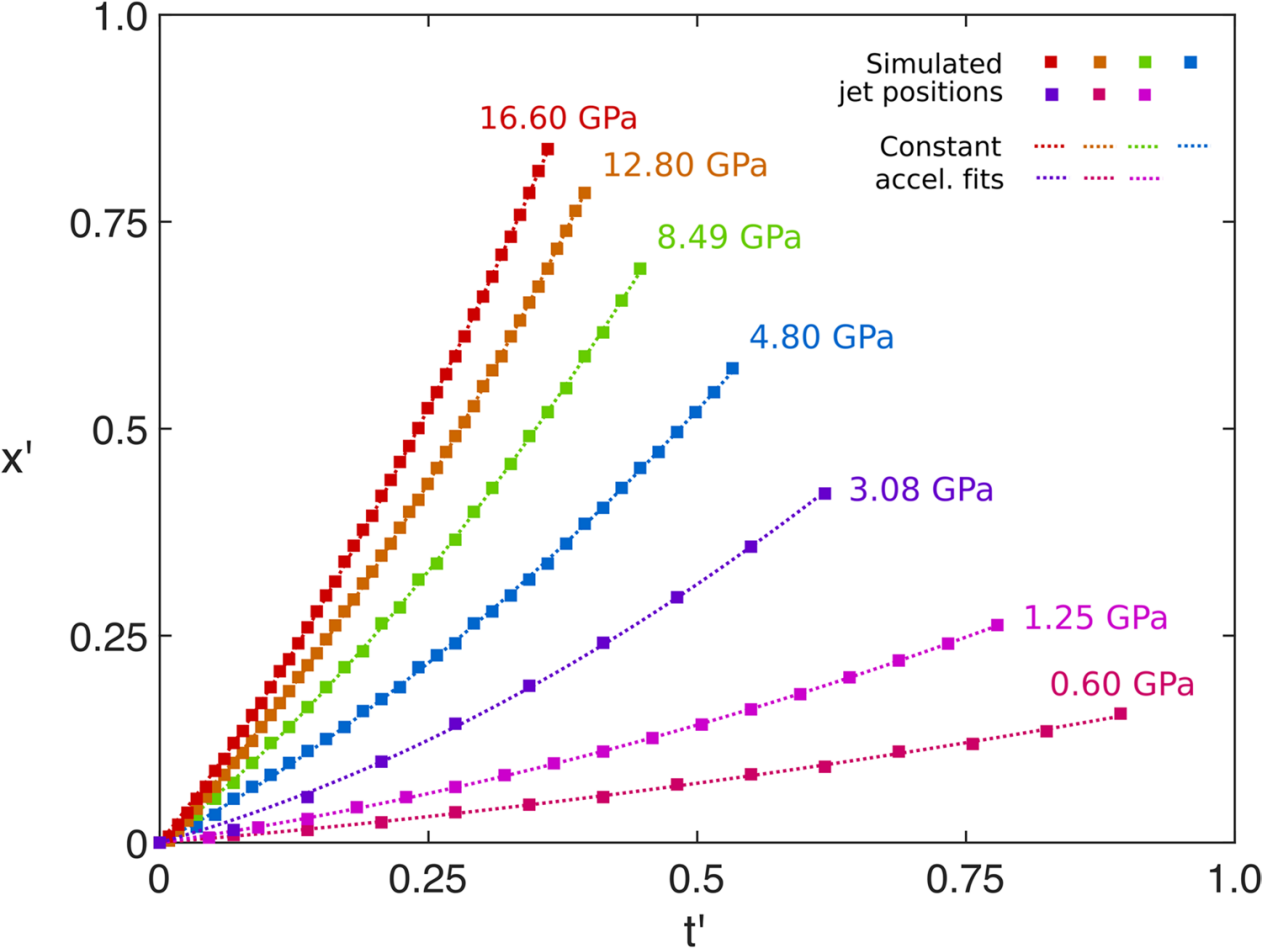
Plots of *x*′ vs. *t*′ extracted from simulations in Hytrac, with constant-acceleration quadratic fits.

**Figure 9:**
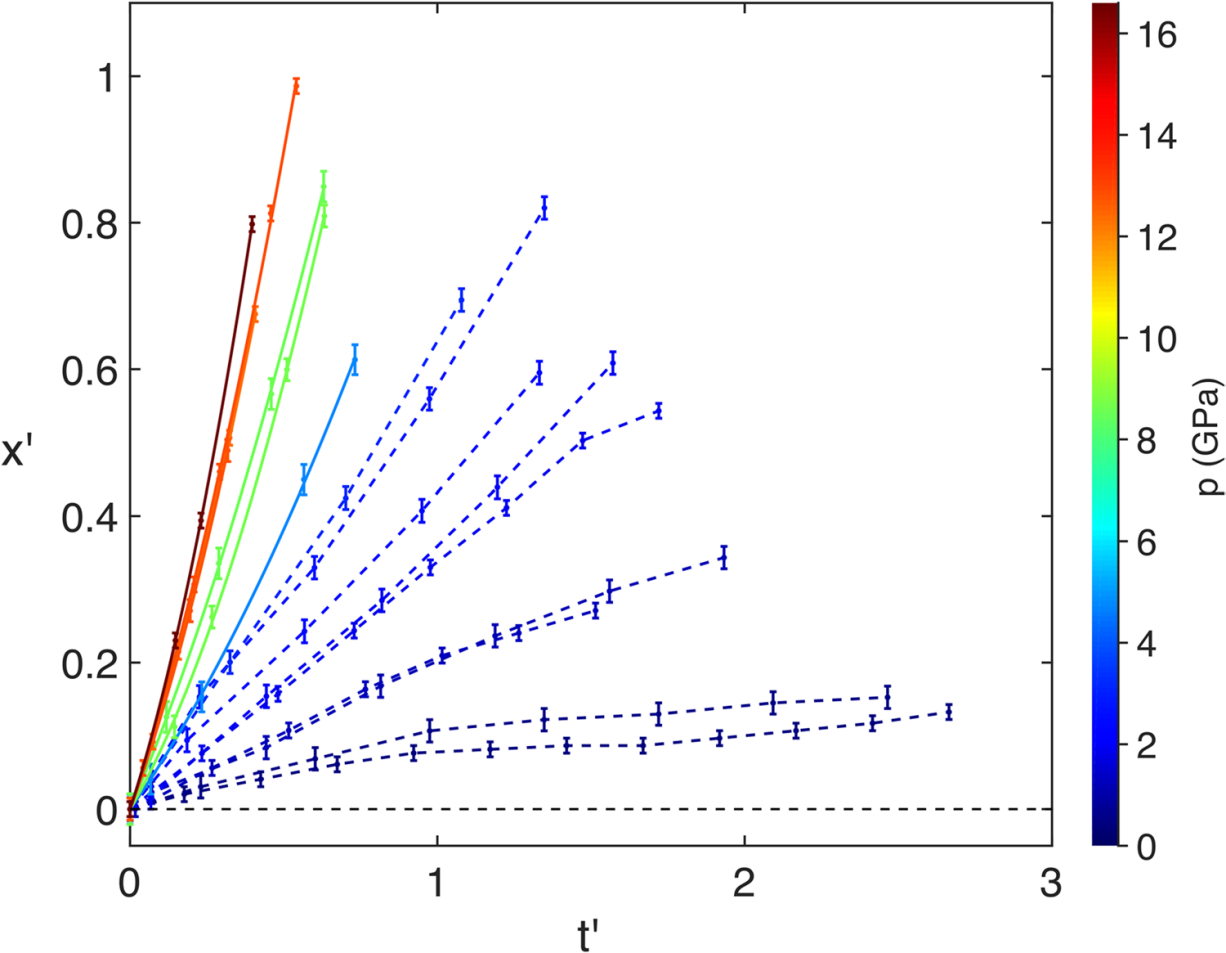
Plots of *x*′ vs. *t*′. The solid lines show a quadratic fit for each interaction and a linear change in the initial shock pressure *p* is represented by the colour bar. The dashed lines show a piecewise linear interpolation of the low-pressure data.

Recalling the right-hand image in Fig. 2(c): after the release of the shock at the upstream cavity interface, the centre of the interface moves downstream with displacement *x*. By introducing a dimensionless interface displacement *x*′ = *x*/*D*_0_, the dimensionless acceleration *a*′, assuming constant acceleration, is determined by fitting the quadratic relationship,

10 $$x{\rm{{\prime} }}=(a{\rm{{\prime} }}/2){t{\rm{{\prime} }}}^{2}+{v{\rm{{\prime} }}}_{0}t{\rm{{\prime} }},$$

where ${v{\rm{{\prime} }}}_{0}$ is the initial dimensionless velocity. This assumption is supported by analysis of *x*′ vs. *t*′ curves extracted from hydrodynamic Hytrac simulations over the full range of shock pressures studied experimentally, shown in Fig. 8. The quadratic fits of the seven curves have *R*^2^ values in excess of 0.99, confirming constant jet acceleration is a characteristic of cavity collapse in fluids. The plots from the experimental data are shown in Fig. 9, where the colour bar represents a linear scaling of *p*. Contrasted to the simulation plots, for the weaker shocks below *p* = 4.80 GPa the curves are not well-fitted by constant acceleration. The trajectories of these displacements are instead shown as dashed lines with piece-wise linear interpolation between the points. This demonstrates the assumption of constant acceleration is not valid in the strength regime.

**Figure 10:**
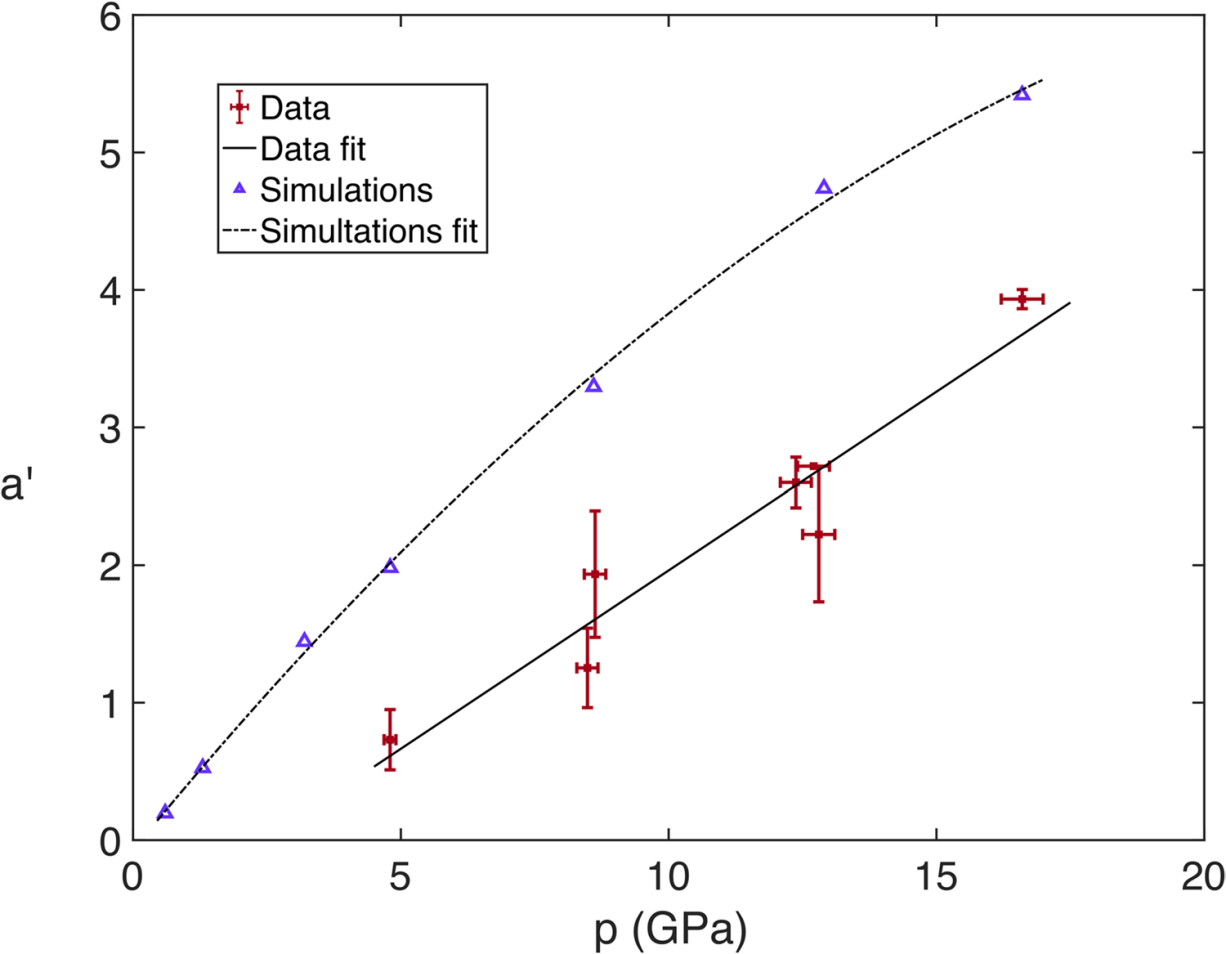
Plots of *a*′ vs. *p*, with the data for the experiments with shock pressures of 4.80 GPa and above. The solid line shows the linear fit of the experimental data and the dashed line shows the quadratic fit for the simulations.

Figure 10 shows the results of the fitted acceleration parameter *a*′ as a function of shock pressure *p*, for both the Hytrac simulations and the data extracted from the radiographs above *p* = 4.80 GPa. The simulation data is best fitted by a quadratic relationship given by,

11 $${a{\rm{{\prime} }}}_{sim}=-0.01{p}^{2}+0.48p-0.07,$$

with an *R*^2^ value of 0.99. However, the experimental data is adequately described by a linear relationship,

12 $${a{\rm{{\prime} }}}_{data}=0.29p-0.93,$$

with an *R*^2^ value of 0.98. Notwithstanding the difference in dimensionality between the simulations and experiments (2D cylindrical vs. 3D spherical), the clearest source of the model deviating from the radiographic results is the lack of a strength model in the simulations. If the effects of strength were to abruptly disappear above the transition to fluid-like behaviour, we would expect the experimental curve to quickly converge with the simulations. Instead we see a consistent offset in the acceleration values, suggesting the effects of strength persist and influence the dynamics of cavity collapse in PMMA at shock pressures in excess of 16.60 GPa. These observations will be investigated in future work using simulations that include a strength model.

## Discussion

There is a visible transition in the radiographs of Figs. 3–5, from strength-dominated to fluid-dominated dynamics with increasing shock pressure. For the lowest shock pressures strength features are dominant in PMMA, with high levels of surface perturbations and damage. In the mid-range of pressures between 1.84 and 3.08 GPa material flow becomes the dominant deformation mechanism, with the the collapse highly symmetric and qualitatively similar to hydrostatic Rayleigh collapse (see Fig. 4). The cavities were observed to collapse above 1.25 GPa, with those at 1.84 GPa and above obeying a power law relationship. The remarkable similarity to the relationship found by Swantek & Austin^25^ suggests that cavity collapse in this regime can at least in part be described in terms of hydrodynamic scaling and points to an underlying universality of shock-induced cavity collapse, even for spherical cavities in solid media and at pressures ratios an order of magnitude greater. Furthermore, the offset between the PMMA data and the gel data can be explained by the differences in sound speed and density, for both the analytic Rayleigh collapse times and the experimental shock-induced data. Hydrodynamic simulations of the experiments, which didn’t include a strength model, predicted a constant acceleration of the driven cavity interface. Analysis of cavity kinematics from the data showed that the motion is not well described by constant acceleration until 4.80 GPa. This indicates that a transition to a hydrodynamic response occurs between 3.08 GPa and 4.80 GPa.

Previous studies identify a change in the *U*_*s*_-*u*_*p*_ Hugoniot equation of state, from a cubic to linear relationship, at a shock pressure of ~2 GPa^44^. Drawing upon Pastine’s equation of state model for polyethylene^57^, Carter & Marsh explain the non-linear region of the curve in terms of the molecular structure of amorphous polymers generally: the forces along the backbone of the polymer chain are an order of magnitude greater than the forces between neighbouring chains. The initial compression of the polymer is thus dominated by the inter-molecular forces in the planes orthogonal to the polymer backbone^46^. By ignoring the spatial dimension along the backbone, the *P*(*V*) relationship is modelled in two dimensions, where *V* is the volume, and then transformed into the *U*_*s*_-*u*_*p*_ plane. This reproduced the observed cubic shape in the low-pressure region of the Hugoniot. Using the same arguments, Carter & Marsh explain that the linear region above the cubic region in the *U*_*s*_-*u*_*p*_ Hugoniot is initiated when the initial shock compression reaches a level at which the inter-molecular forces approach a significant value with respect to the forces along the chain. At this point the polymer behaves in the expected manner of a shocked isotropic solid^46^.

It is interesting to note that different transitions have been observed within this pressure region in other experiments on the shock response of PMMA: (i) Lacina *et al*.^44^ observed a change in the characteristic relaxation time constant in the rounding of the *u*_*p*_ wavefronts, which increased to a maximum value at 1 GPa before decreasing rapidly until 2 GPa; (ii) A sudden increase in the dielectric constant above 2 GPa was observed by Hauver^58^ and Graham^59^; (iii) A similar increase in the temperature of shocked PMMA at this pressure was observed by Bloomquist & Sheffield^60,61^; (iv) Jordan *et al*.^62^ measured the dependence of the strength on impact pressure, combining their data with that of an earlier Millet & Bourne study^63^. They observed the start of an approximate plateau in shear strength with increasing shock pressure above 2 GPa, indicative of a transition to hydrodynamic behaviour^15^. Although the precise mechanisms which underlie these phenomena have yet to be established, these studies point to a significant transition in the shock response of PMMA at shock pressures of ~2 GPa. In the present work, this transition is manifest as a change in the collapse characteristics of cavities in PMMA, from a strength-dominated mode to a hydrodynamic mode in which the collapse energy is either distributed throughout the cavity periphery, or concentrated within the jet. Interestingly, this is the approximate pressure at which the collapse times begin to fall on the power-law relationship curve (1.84 GPa and above), which extends into the hydrodynamic regime, suggesting this may correspond to a change in collapse dynamics and the onset of a transition to fluid-like behaviour. This transition demarcates distinct regimes of dynamic material behaviour differing in how shock energy is internalised, which furthermore can play an important role in driving processes that depend upon the precise distribution of energy.

One example of the importance of shock energy distribution lies in the impact initiation of energetic materials. Mesoscale simulations of shock-cavity interactions in HMX by Rai *et al*.^64^ revealed similar collapse dynamics as observed in the present study: (i) the presence of perturbations and shear localisation in the weak shock regime; (ii) intermediate strength shocks producing weak jetting; and (iii) strong shocks generating pronounced jets and high levels of vorticity and mixing after collapse. The results also predicted a dependence between shock loading pressure and the size and shape of hot spots, key to yielding a sustained reaction, with the reaction threshold further linked to the ratio of shock strength to the yield stress of HMX. In the present work, the presence of tori in the experiments for shock pressures 8.49 GPa and above also indicate high levels of vorticity (see the tori in the images of Fig. 5(b,c)). This phenomenon, which has been observed in gaseous^21,22^ and liquid^24^ media, indicates a high level of material mixing, which is necessary for prompt, non-diffusive mass transport of reactant species^65^.

## Summary

High-speed radiography was used to reveal the time evolution of sub-surface features during shock-induced cavity collapse in a solid. These included surface perturbations and crack propagation for weaker shocks, and jet and tori formation for strong shocks. A transition from brittle fracture to material flow was observed, and, consistent with studies that have looked at shock-cavity interactions in liquids, analysis of the normalised projected cavity area vs. dimensionless collapse time revealed a power law relationship between the collapse time and shock pressure at 1.84 GPa and above. Surprisingly, this relationship holds across the shock regimes, from strength-dominated to hydrodynamic. Strength-free simulations performed in Hytrac exhibited constant jet acceleration during hydrodynamic collapse, suggesting this to be a common characteristic of fluids. The present experimental measurements however reveal more complex collapse kinematics, with the onset of jet formation and constant acceleration behaviour occurring at pressures in excess of 4.80 GPa. Taken alone, these observations imply a complete loss of strength at this critical pressure. The persistence of an offset between the measurements and the ideal fluid behaviour rather indicates the prevailing effects of strength well above this transition.

We have presented previously unobserved features of shock-induced cavity collapse in a solid medium. The results showcase the latest developments in UHS synchrotron imaging capabilities, which allow sub-surface dynamics of transient events to be captured to an unprecedented level of detail. The quality of the data and its broad scope will be essential for the development and improvement of new models governing the dynamic behaviour of solids in both the strength and hydrodynamic regimes, as well the less-explored transition regime. Specifically, we expect the direct observations of hydrodynamic phenomena such as jet formation, impact and tori formation to be an invaluable resource for research into medical treatments where cavity collapse plays a central role. Additionally, new details on the richness and complexity of strength-mediated collapse will help guide the design of improved materials for harnessing and releasing energy.

## Data Availability

The datasets used in the current study are available from FigShare on https://figshare.com/authors/Emilio_Escauriza/8718576.
